# Screening and Identification of Human Endogenous Retrovirus-K mRNAs for Breast Cancer Through Integrative Analysis of Multiple Datasets

**DOI:** 10.3389/fonc.2022.820883

**Published:** 2022-02-16

**Authors:** Yongzhong Wei, Huilin Wei, Yinfeng Wei, Aihua Tan, Xiuyong Chen, Xiuquan Liao, Bo Xie, Xihua Wei, Lanxiang Li, Zengjing Liu, Shengkang Dai, Adil Khan, Xianwu Pang, Nada M. A. Hassan, Kai Xiong, Kai Zhang, Jing Leng, Jiannan Lv, Yanling Hu

**Affiliations:** ^1^Guangxi Clinical Center for AIDS Prevention and Treatment, Chest Hospital of Guangxi Zhuang Autonomous Region, Liuzhou, China; ^2^Institute of Life Sciences, Guangxi Medical University, Nanning, China; ^3^Department of Chemotherapy, The Affiliated Tumor Hospital of Guangxi Medical University, Nanning, China; ^4^Guangxi Medical University School of Information and Management, Nanning, China; ^5^Basic Medical College of Guangxi Medical University, Nanning, China; ^6^Cancer Hospital, Guangxi Medical University, Nanning, China; ^7^Guangxi Collaborative Innovation Center for Biomedicine (Guangxi-ASEAN Collaborative Innovation Center for Major Disease Prevention and Treatment), Guangxi Medical University, Nanning, China; ^8^Guangxi Key Laboratory of Translational Medicine for Treating High-Incidence Infectious Diseases With Integrative Medicine, Guangxi University of Chinese Medicine, Nanning, China; ^9^Center for Genomic and Personalized Medicine, Guangxi Key Laboratory for Genomic and Personalized Medicine, Guangxi Collaborative Innovation Center for Genomic and Personalized Medicine, Guangxi Medical University, Nanning, China

**Keywords:** human endogenous retrovirus-K, breast cancer, datasets, DESeq2, expression

## Abstract

**Objective:**

Human endogenous retroviruses (HERVs) make up 8% of the human genome. HERVs are biologically active elements related to multiple diseases. HERV-K, a subfamily of HERVs, has been associated with certain types of cancer and suggested as an immunologic target in some tumors. The expression levels of HERV-K in breast cancer (BCa) have been studied as biomarkers and immunologic therapeutic targets. However, HERV-K has multiple copies in the human genome, and few studies determined the transcriptional profile of HERV-K copies across the human genome for BCa.

**Methods:**

Ninety-one HERV-K indexes with entire proviral sequences were used as the reference database. Nine raw sequencing datasets with 243 BCa and 137 control samples were mapped to this database by Salmon software. The differential proviral expression across several groups was analyzed by DESeq2 software.

**Results:**

First, the clustering of each dataset demonstrated that these 91 HERV-K proviruses could well cluster the BCa and control samples when the normal controls were normal cells or healthy donor tissues. Second, several common HERV-K proviruses that are closely related with BCa risk were significantly differentially expressed (*p*_adj_ < 0.05 and absolute log2FC > 1.5) in the tissues and cell lines. Additionally, almost all the HERV-K proviruses had higher expression in BCa tissue than in healthy donor tissue. Notably, we first found the expression of 17p13.1 provirus that located with TP53 should regulate TP53 expression in ER+ and HER2+ BCa.

**Conclusion:**

The expression profiling of these 91 HERV-K proviruses can be used as biomarkers to distinguish individuals with BCa and healthy controls. Some proviruses, especially 17p13.1, were strongly associated with BCa risk. The results suggest that HERV-K expression profiles may be appropriate biomarkers and targets for BCa.

## Background

Breast cancer (BCa) is the most common cancer diagnosed in women and is one of the three most common cancers worldwide (1, 2).The heterogeneity, complex etiology, diverse gene mutations, and different clinical manifestations of BCa denote that all kinds of internal and external risk factors may participate in BCa pathogenesis. In addition to external and internal risk factors, genetics and epigenetics can initiate signaling pathways in BCa through the regulation of different genes (3–5).

Previous studies have shown that human endogenous retrovirus (HERV) can stimulate tumor cell proliferation and avoid apoptosis, which is one of the most important factors for tumor progression (6). HERV complete proviruses and genes have been inserted into the host genome, account for approximately 6%–8% of the human genome, and have resided in the human genome for millions of years (7, 8). HERVs are divided into three groups based on exogenous sources: class I (Gammaretrovirus and Epsilonretrovirus-like), class II (Betaretrovirus-like), and class III (Spumaretrovirus-like) (9, 10). Most HERVs have become dysfunctional because of the accumulation of multiple nonsense mutations, and some are still active and may play a role in human disease. The most recent proviruses to invade the human genome ARE the HERV-K (HML-2) family (11–13). Approximately 90 HERV-K proviruses and many smaller elements have been detected in the human genome (14). HERV-K transactivation has been observed in a variety of human cancers, such as leukemia (15), lymphoma (16), BCa (17), and melanoma (18). For instance, the expression of HERV-K envelope protein (Env) in BCa is higher than that in nonmalignant BCa, and some anti-HERV-K-specific monoclonal antibodies can effectively inhibit the growth of BCa cells and induce their apoptosis *in vitro* and *in vivo* (17). In addition, HERV-K has multiple copies in the human genome, and some complete open reading frames can be found in HERV-K proviruses. Although HERV-Ks often demonstrate important roles on BCa, studies on the transcriptional activity of this provirus across the human genome are still lacking.

Some investigations have addressed the association between HERV-K mutation and BCa risk. Montesion et al. (19) identified two unique binding sites in each 5′ long terminal repeats that appear to be associated with the induction of promoter activity for BCa. Mark et al. (20) found that Xq21.33 mutated by gene conversion in a subset of African populations is associated with human BCa. Many studies focused on the activity of HERV-K genes and the function on BCa and found that HERV-K env, gag, and pol activities are associated with tumor size, tumor stage, and survival (21, 22). However, HERV-Ks are embedded in many human genome loci and have different sequences and functions. Therefore, determining the transcriptional profiles of these HERV-Ks across the human genome is essential to clarify their potential risk for BCa.

## Materials and Methods

### RNA-seq Data of BCa and Normal Samples

The RNA sequences of BCa and normal samples were downloaded from the National Center for Biotechnology Information (NCBI) Sequence Read Archive (SRA) database before October 2021. Studies were screened according to the following inclusion and exclusion criteria: (1) the datasets should be samples of BCa and healthy controls; (2) for each dataset, at least three samples were for cancer and controls, respectively; (3) all datasets were raw data obtained by high-throughput sequencing. Datasets treated with drugs or disturbed through gene expression interference were removed. Nine datasets were included in this study. The details of each dataset are shown in **Supplementary Table S1**.

The datasets included in this study contain RNA sequencing data from nine laboratories. The datasets comprised 199 clinically aggressive BCa samples, 11 normal breast controls from healthy donors, and 109 control tissues adjacent to tumor tissues. Among the 199 BCa samples, 72 ER+ Bca, 13 HER2+ BCa, and 59 triple-negative BCa (TNBC) can be abstracted from the nine datasets. Additionally, 44 samples of BCa cells (MCF7, ZR751, MB361, UACC812, SKBR3, AU565, HCC1954, MB231, MB436, MB468, and HCC1937) and 8 normal control cells (76NF2V and MCF10A) were downloaded from NCBI SRA. The raw sequencing data are publicly available in the NCBI biorepository (https://www.ncbi.nlm.nih.gov/). We downloaded the data using the NCBI SRA Toolkit (**Supplementary Table S1**). Trimmomatic3 (23) was used to remove adaptors and low-quality reads. FastQC (v0.11.3) was then applied to confirm the quality of the raw reads and trimmed low-quality reads of each sample.

### Expression Profiles of HERV-K Proviruses Mapped From Raw RNA-seq Data

The identified HERV-K proviruses are not well annotated in public databases. Therefore, we downloaded the FASTA sequences of the 91 HERV-K proviruses deposited in NCBI (GenBank ID JN675007–JN675097) (24). The FASTA sequence files of the 91 HERV-K proviruses were matched with the GRCh38 cDNA FASTA files downloaded from Ensembl to determine the transcriptional profiles of these human genes. In this study, the expression of HERV-K was analyzed by defining the entire proviral sequence as a single transcript but not the individual potential spliced transcripts. Salmon software (25), capable of fast and bias-aware quantification of transcript expression, was used to create an index database and a count matrix over the full human transcriptome joining with the HERV-K file. Index building, for example, was written as salmon index -t GRCh38_HERVK_trans.fa -i trans_HERVK_index, and count matrix computing was expressed as salmon quant -i./trans_HERVK_index/-l A -1 GSE96860_1P.fastq -2 GSE96860_2P.fastq -o./salmon/SRR/–validate Mappings. Finally, we selected the read counts matrix assigned to the 91 HERV-K loci.

### Statistical Analysis of HERV-K Expression Between Tumors and Controls

Transcript abundance reads were evaluated by the Salmon package in R software (version 4.1.1) using tximport (26). In view of the different sample sequencing depths, transcript abundance was normalized for each dataset using the R Bioconductor package, DESeq2 (27). This method allowed us to confirm the normalization of HERV-K expression across the proviral loci by sample. The differential expression of HERV-K was defined by comparing BCa and healthy donors or adjacent normal tissues or between BCa cells and normal breast cells. Additionally, the different expression of HERV-K was further analyzed between BCa subtypes (ER+, HERS+, and TNBC) and normal controls. If the *p*-adjusted value (*p*_adj_) was under 0.05 and the absolute value of the log2 fold change (|log2FC|) was greater than 1.5, then these HERV-K proviruses had significant differential expression (24, 28).

## Results

### Characteristics of HERV-K Expression Signatures Across Different Studies Among Nine Datasets

All raw sequencing data and clinic information downloaded from the Gene Expression Omnibus and NCBI SRA were mapped to the 91 HERV-K provirus indexes by Salmon software. After the transcripts from the RNA-seq data were quantified, dataset GSE111842 was removed because the number of HERV-K reads in this dataset was almost zero. DEseq2 software was used to normalize the remaining eight datasets. DEseq2 was used to compare the digital expression between BCas and controls in the eight datasets separately to obtain the differentially expressed HERV-Ks in each dataset. Except GSE183947, several different proviruses were detected in each dataset (*p*_adj_ < 0.05 and |log2FC|). The detailed information of the eight datasets and the number of differentially expressed HERV-Ks identified from each dataset are shown in **Supplementary Table S2**. The results indicated that the total number of different proviruses was higher in BCa tissues than in normal tissues from healthy donors, but few different proviruses were discovered between tumors and adjacent normal tissues. Additionally, the proviruses indicated opposite expression profiles under different control cells (76NF2V and MCF10A).

### Differential Expression Levels of HERV-Ks in Tumor Cells Compared With Healthy Control Cells

According to the cell definitions of Chappell et al. (29) and Subik et al. (30), we selected three types of BCa cell lines (ER+, HER2+, and TNBC). Therefore, datasets GSE96860, MCF7 (ER+), AU565 (HER2+), MB231 (TNBC), and MB468 (TNBC) were included as cancer cell lines, and MCF10A and 76NF2V were selected as the normal healthy cell lines. Tumor cells (MB231 and BRCA1−/−) and normal healthy cells (MCF10A) in dataset GSE171957 were considered. After the normalized data were analyzed by DEseq2 software, the general cluster of HERV-K expression profiles across all samples was visualized in the HERV-K expression heatmap (**Supplementary Figures S1**–**S3**). The results showed that the expression of HERV-K proviruses could well cluster cancer and control cells separately. This finding indicates that the expression of these 91 HERV-Ks can be used as potential markers for BCa cells.

**Figure 1:**
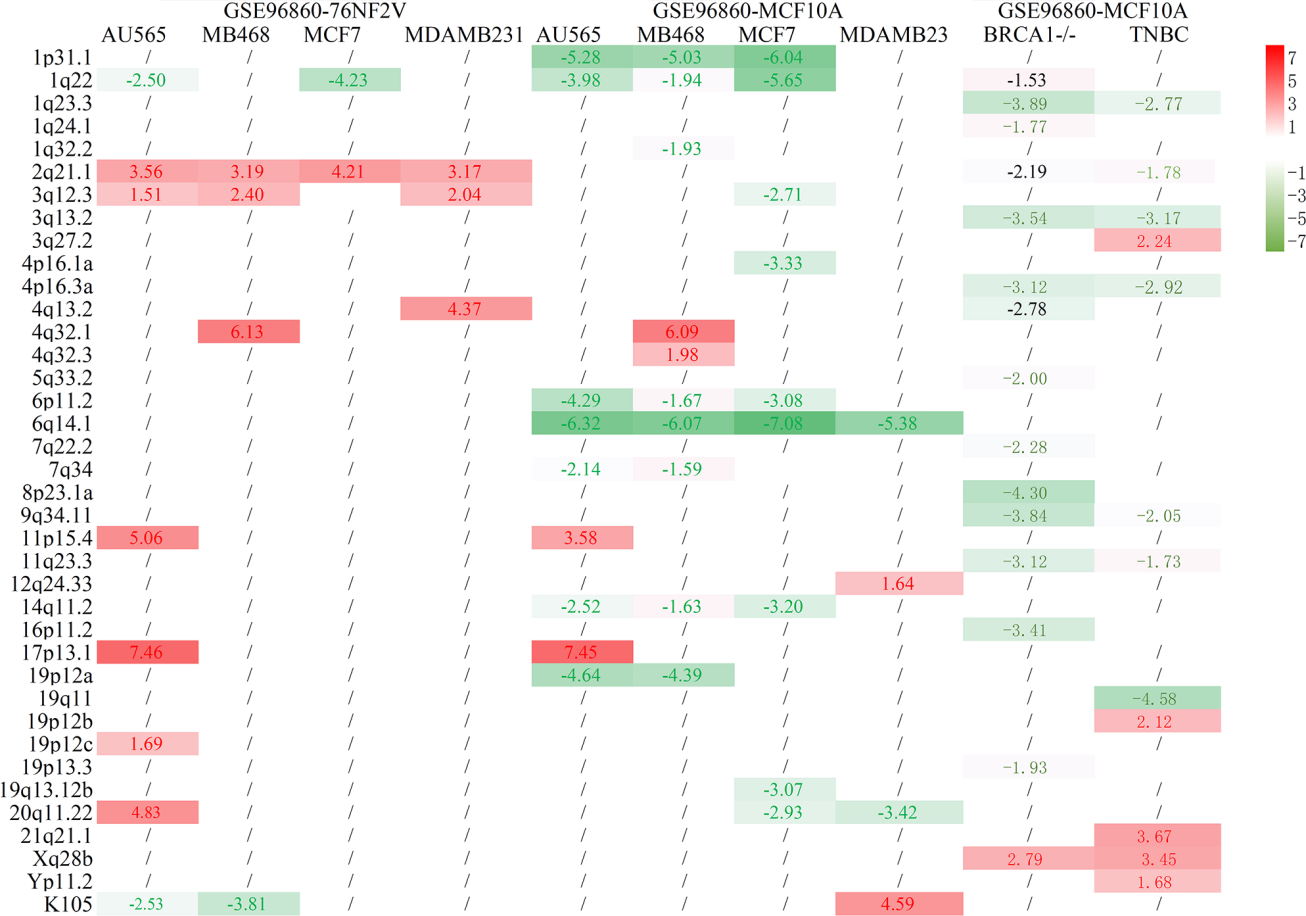
Statistical results of BCa and normal control cells. The row is the loci of HERV-K proviruses, and the column is the cell comparison.

The differential proviral expression between BCa and control cells was calculated. Compared with normal 76NF2V cells in dataset GSE96860, most of the differentially expressed proviruses were upregulated in BCa cells. However, almost all remarkably different proviral loci were downregulated compared with normal MCF10A cells (**Figure 1**). This condition was also reflected in dataset GSE171957. The main reason could be the higher expression of HEVR-K proviruses in MCF10A cells than in 76NF2V cells (**Supplementary Figure S4** and **Supplementary Table S4**). 1q22 was downregulated in AU565 and MCF7 cells based on the comparison of both normal cells. 2q21.1 was significantly expressed in all groups in the normal cell line, 76NF2V (*p*_adj_ < 0.05 and |log2FC| >1.5). Compared with normal MCF10A cells, the provirus in locus 6q14.1 was expressed much more in BCa cells than in control cells (**Figures 1B–E**) with *p*_adj_ < 3.03 × 10^−5^ and |log2FC| >5 across all conditions. Five proviruses (1p31.1, 1q22, 6p11.2, 6q14.1, and 14q11.2) were significantly differentially expressed in AU565 (HER2+), MB468 (TNBC), and MCF7 (ER+) cells (*p*_adj_ < 0.05 and |log2FC| >1.5). Locus 17p13.1 located in the TP53 gene had a much higher expression in AU565 (HER2+) cells than in 76NF2V (log2FC = 7.46) and MCF10A cells (log2FC = 7.45). These results indicate that the expression of HERV-K proviruses has high heterogeneity in different cells.

### Abnormal Overexpression of HERV-Ks Discovered in BCa Tissues Compared With Control Tissues From Healthy Donors

**Figure 2:**
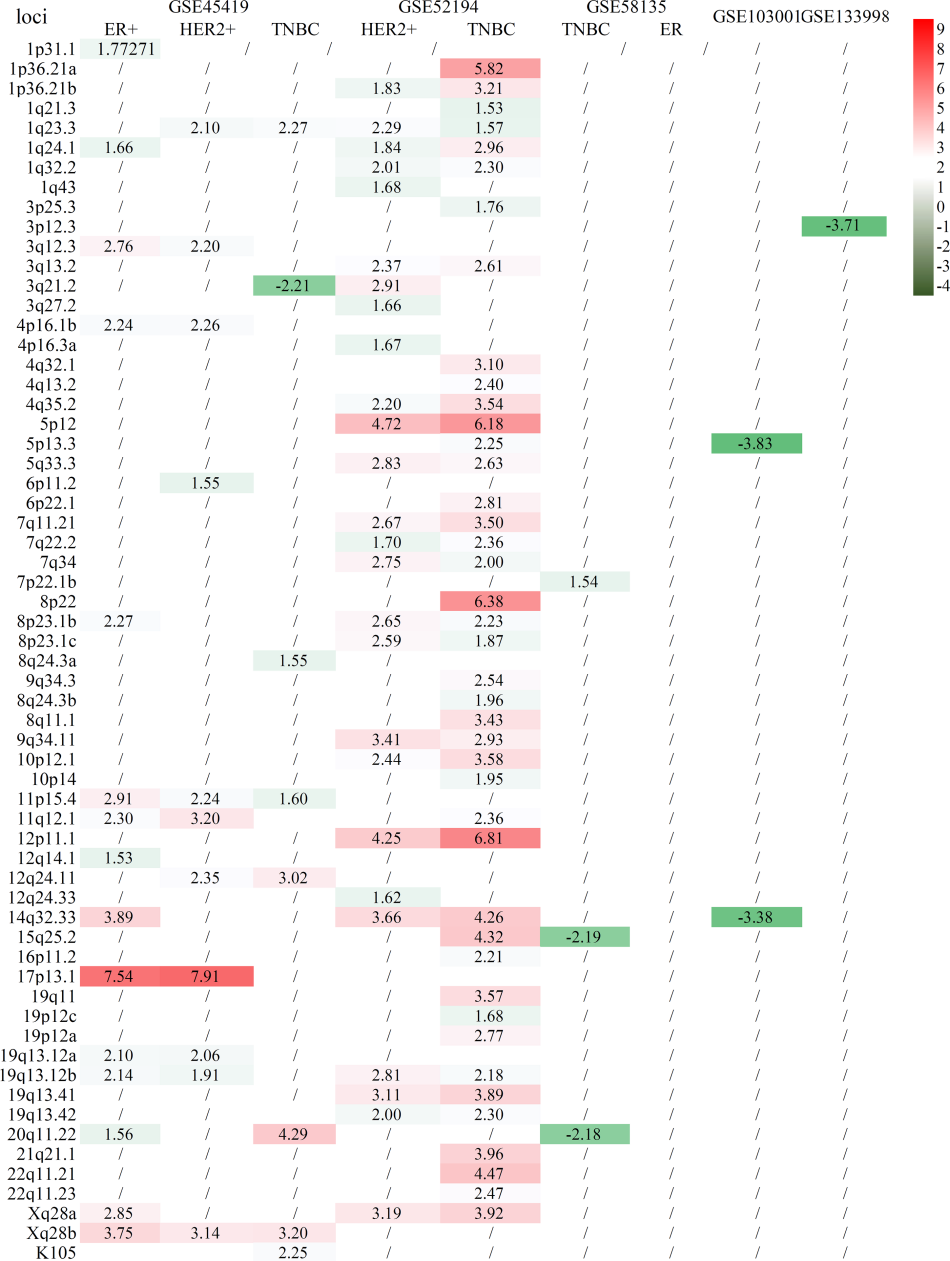
Statistical comparison of several BCa cell lines to normal controls from healthy donors. The row is the loci of HERV-K proviruses, and the column is the comparison of tumor subtypes to normal tissue.

Six tissue datasets of raw sequencing data, namely, GSE45419, GSE52194, GSE58135, GSE133998, GSE103001, and GSE183947, were downloaded from the NCBI SRA database. In these datasets, the controls of two datasets (GSE45419 and GSE52194) were benign epithelial cells or normal human breast organoids from healthy donors, and the other four datasets (GSE58135, GSE133998, GSE103001, and GSE183947) were obtained from adjacent breast tissues. The samples were categorized as ER+, HER2+, and TNBC BCa if the tumor types could be mined from the clinical information. After the clustering of the datasets for ER+, HER2+, and TNBC BCa samples and controls, almost all cancer samples and normal controls could be separated in two datasets (GSE45419 and GSE52194). Most of the expressed proviral loci were higher in BCa tissues compared with controls as demonstrated by the heatmap of HERV-K expression profile (**Supplementary Figure S5**). However, except for GSE183947, the clustering of normalized data showed that the BCa and control samples were mixed together in all datasets if the control samples used were normal tissues adjacent to cancer tissues (**Supplementary Figures S6**–**S9**). Differentially expressed proviruses between the BCa tissues and control samples from each dataset were analyzed. Among all datasets with tissue sequencing, the normal control samples from GSE45419 and GSE52194 were healthy donors without BCa, and the controls from other datasets were adjacent normal tissue. The analysis results showed that many different abnormal HERV-Ks were found in the two datasets when the control samples were from normal healthy donors, but few different HERV-Ks were found in the other five datasets (**Figure 2**). Almost all the remarkably different proviruses in two datasets (GSE45419 and GSE52194) had higher expression in the BCa samples than the control samples. 19q13.12b had a much higher expression in the ER+, HER2+, and TNBC groups (*p*_adj_ < 0.05, |log2FC| >1.5) in datasets GSE45419 and GSE52194. Locus 1q23.3 was found to be the common abnormal locus with abnormally higher HERVK expression in the TNBC and HER2+ groups of two datasets (GSE45419 and GSE52194). 17p13.1 was expressed higher in the ER+ and HER2+ groups in dataset GSE45419, and 3q12.3 was remarkably overexpressed in ER+ BCa tissue.

A further analysis of the composition of the sample in GSE183947 revealed that the breast tumor samples in this dataset included 15 metastatic or 15 unmetastatic samples. These results indicate that HERV-Ks are expressed in BCa tissues and adjacent control samples, but if the tumor tissues are in the progress of metastasis, HERV-Ks will have different expression levels (**Supplementary Figure S10**).

### Expression of 17p13.1 Provirus Was Closely Related to the Expression of Tumor Protein p53

**Figure 3:**
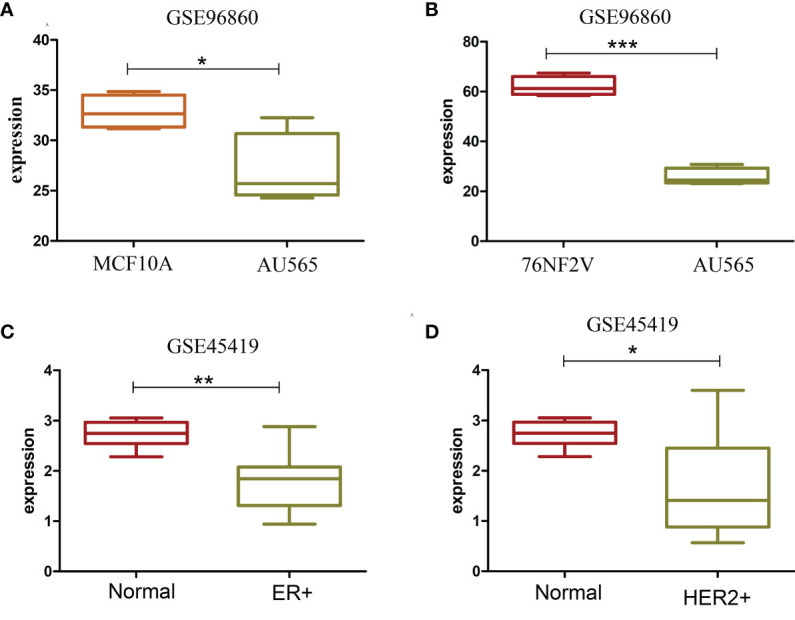
Box plot of TP53 with significantly different expression in two datasets. **(A)** TP53 expression in MCF10A and AU565 (HER2+) cells. **(B)** TP53 expression in 76NF2V and AU565 (HER2+) cells. **(C)** TP53 expression in control and ER+ BCa tissues. **(D)** TP53 expression in control and HERS2+ BCa. *P < 0.05; **P < 0.01; ***P < 0.001.

17p13.1 located in tumor protein p53 (TP53) had a higher expression in the ER+ and HER2+ BCa samples than in normal cells and tissues. TP53 is a very important tumor suppressor protein. TP53 expression across several groups was analyzed to clarify the relation between 17p13.1 and TP53. Contrary to the expression of 17p13.1 provirus, the expression of TP53 was lower in AU565 than in MCF10A (*p* = 0.025) and 76NF2V (*p* < 0.001). 17p13.1 provirus in AU565 was expressed in each sample, but the control MCF10A had zero expression in all samples. In dataset GSE45419, a lower expression was found in the ER+ (*p* = 0.001) and HER2+ (0.012) normal samples. Compared with the controls in dataset GSE45419, when the 17p13.1 provirus was expressed, TP53 had a low expression in the TNBC samples (**Supplementary Table S3**). Additionally, in dataset GSE52194, although no remarkable overexpression was found in the HER2+ BCa samples, the log2FC was 1.838. TP53 expression was lower in the HER2+ BCa samples than the normal samples (log2FC = −0.957) (**Figure 3**). The results indicate that the expression of 17p13.1 provirus is closely related to TP53 expression in the ER+, HER2+, and TNBC BCa samples.

## Discussion

HERV-K expression is frequently inhibited in normal cells from healthy adults, but their mRNA expression increases in tumor cells (31). However, the expression of multiple HERV-K copies in the human genome for BCa lacks a clear description. Previous reports focused on the relation of total HERVK expression with BCa but not on the whole-genome details of the loci. In this study, we mined the literature, downloaded raw sequencing data from the NCBI SRA database, and used Salmon software to map the 91 HERV-K indexes. The healthy cell lines and control breast tissues from healthy donors were compared, and the results showed that the 91 HERV-Ks could distinguish cancer and control samples. However, if the controls were from adjacent normal tissues, the tumor and control samples cannot be clustered clearly according the expression of the 91 HERV-Ks. Second, two controls (normal samples from healthy donors and para-carcinoma tissue) were tested to analyze the different expressing provirus between tumor and control tissues. The results showed that several HERV-K proviruses, such as 17p13.1, 19q13.12b, and 1q23.3, had remarkably different expression levels between BCa and controls across several datasets. Third, the expression of HERV-K proviruses showed high heterogeneity in different cells and cancer types. Additionally, most of the remarkably expressing HERV-Ks were increased in BCa compared with normal tissue controls.

HERVs are a substantial part of the human genome, but most of them remain transcriptionally silent. In this study, 91 HERV-Ks, whose entire proviral sequence was defined as a single transcript, were analyzed. According to all the heatmap clustering of all the datasets, except the controls from para-carcinoma tissues, the 91 HERV-Ks can well split cancer and control samples. The expression profiles of the 91 HERV-K proviruses can be used as biomarkers to cluster BCa and healthy control samples. In BCa, HERV-K expression is about 26% in tumor tissues and 18% in adjacent normal breast tissues (32). Many investigations discovered that HERV-K expression in the blood is elevated at the early stage of BCa and further increases in patients who are at risk of developing a metastatic disease (33). Additionally, HERV-K can be a novel target for BCa immunotherapy (17, 34, 35). Kaplan et al. (20) showed the possible increased prevalence of Xq21.33 provirus in post-menopausal Nigerian women with BCa. In our studies, we also discovered that the expressing profile of 91 HERV-K proviruses can separate cancer samples between metastasis and adjacent normal breast tissues. Golan et al. (32) also found a remarkable correlation between HERV-K RT expression and the poor prognosis of disease-free patients who continued to develop the disease. Saini et al. (36) indicated that several genes in the HERV-K family, including env, gag, and np9 mRNA expression levels, are increased in BCa cells and can be used as biomarkers for early BCa diagnosis. The expression of HERV-K env gene was related to tumor size, tumor stage, and lymph node metastasis. Moreover, compared with those with moderate or low HERV-K env expression, the population with high HERV-K env expression has increased overall survival (37). Additionally, HERV-K gag mRNA also has a higher expression in patients with metastatic BCa than those with benign tumors (33). Saini et al. (36) uncovered the T-cell recognition of HERVs in myeloid malignancies and indicated that HERVs are potential targets for immunotherapy.

In about half of all human cancers, the tumor suppressor gene, *TP53*, located at 17p13.1, is lost or mutated. In our study, the expression of a particular provirus was found to be remarkably higher in BCa than in normal controls. The functionality of p53 on BCa has been confirmed by many studies. The loss of p53 protein function influences the cell cycle checkpoint control and apoptosis, as well as the regulation of other important stages of metastatic progressions, such as cell migration and tissue invasion (38). Primary BCa tumors with loss of TP53 copies have a poorer prognosis and a higher chance of metastasis (38). In BCa, loss of heterozygosity on 17p is a frequent event, and is the *p53* gene on 17p13.1 is a likely target (39). Sequence aligning by BLAST showd that TP53 is mapped to 1,168,421–1,187,490 bp in 17p13.1, and HERV-K (JN675075.1) is located in 1,556,337–1,563,901 bp. In our study, we found that the expression of 17p13.1 provirus was closely related with TP53 expression in the ER+, HER2+, and TNBC BCa cells. Runnebaum et al. (40) found that p53 transdominantly suppresses the tumor formation of human BCa cells mediated by retroviral bulk infection without marker gene selection. However, the mechanism of 17p13.1 provirus on TP53 expression needs to be verified.

In this study, except for 17p13.1, multiple other loci of HERV-K proviruses were related to BCa. 11p15.4 was upregulated in all cancer types in dataset GSE45419 and AU565 cell lines. Montesion et al. (19) indicated that 11p15.4 provirus displays increased activity in almost all human BCa cell lines. Based on the sequences of 2,504 individuals from the 1,000 Genomes Project, they also discovered that the active form of the 11p15.4 site is polymorphic within the human population. León et al. (41) detected that the BCa-associated gene 3 (BCA3, AKIP1) is located on 11p15.4. This gene can regulate the effect of the cAMP-dependent protein kinase signaling pathway on the NF-kappa-B activation cascade (42). In our study, 3q12.3 was remarkably overexpressed in ER+ BCa tissue and cells. This result was similar to the results of Montesion et al. (19).

BCa is a heterogeneous disease with different characteristics in distinct histological, molecular, and clinical phenotypes. In our study, the expression of HERV-K proviruses had high heterogeneity in different cells and cancer types. Johanning et al. (43) reported that HERV-K *env* expression depends on the BCa subtype; it was detected in normal breast tissues and was remarkably upregulated in basal BCa subtypes.

However, the present investigation only focused on the expression of 91 HERV-K proviruses from the entire proviral sequences of BCa and control samples. In addition, the *HERV-K* genes such as env and gag are important targets that affect BCa progression. Therefore, future investigation is needed to explore the gene function of particular HERV-K proviruses across the whole genome and provide targets for immunotherapy. Lastly, the expression of HERV-K proviruses had high heterogeneity in different cells and cancer types. More samples are needed to further verify the correlation between HERV-K expression and BCa.

## Conclusion

The current investigations provide many evidences that the expression profiles of HEVR-K proviruses can be a useful biomarker for BCa. Several HERV-K proviruses are overexpressed in BCa as compared with normal breast controls. The large difference in the expression profiles of HERV-K proviruses indicated that HERV-K expression could be an intriguing target of a tumor-specific antigen for BCa. Future explorations are needed to investigate the differential expression of *HERV-K* genes to use HERV-K expression as a tool for disease stratification and immunotherapy. The expression of 17p13.1 provirus could regulate TP53 expression and BCa progression, especially ER+ and HER2+ BCa.

## Data Availability Statement

The original contributions presented in the study are included in the article/**Supplementary Material**. Further inquiries can be directed to the corresponding authors.

## Author Contributions

YH and JNL conceived the study. AT, XC, and KZ downloaded the data. XL, BX, XW, LL, ZL, AK, XP, and YH analyzed the data. YH, HW, and YFW wrote the manuscript. YZW, SD, NH, and KX drew the figures. JL participates in the conception and revision of the article, and provided good revision suggestions. All authors have reviewed the manuscript. All authors contributed to the article and approved the submitted version.

## Funding

Outstanding Young Talents Training Program of Guangxi Medical University and Guangxi Key Research and Development Program (No. GuikeAB20059002). Guangxi Key RESEARCH and development Program: based on high-throughput sequencing to explore the host origin of coronavirus and other respiratory viruses (GuikeAB20059002).

## Conflict of Interest

The authors declare that the research was conducted in the absence of any commercial or financial relationships that could be construed as a potential conflict of interest.

## Publisher’s Note

All claims expressed in this article are solely those of the authors and do not necessarily represent those of their affiliated organizations, or those of the publisher, the editors and the reviewers. Any product that may be evaluated in this article, or claim that may be made by its manufacturer, is not guaranteed or endorsed by the publisher.

## Supplementary Material

Supplementary Figure S1 Heatmap of tumor and control 76NF2V cells in dataset GSE96860. In all the legends in each cluster, N denotes normal controls, and C denotes tumor samples. **(A)** Comparison of AU565 and 76NF2V; **(B)** comparison of MB468 and 76NF2V; **(C)** comparison of MCF7 and 76NF2V; **(D)** comparison of MDAMB231 and 76NF2V.

Supplementary Figure S2 Heatmap of tumor and control MCF10A cells in dataset GSE96860. In each cluster, N denotes the normal controls, and C denotes the tumor samples. **(A)** Comparison of AU565 and MCF10A; **(B)** comparison of MB468 and MCF10A; **(C)** comparison of MCF7 and MCF10A; **(D)** comparison of MDAMB231 and MCF10A.

Supplementary Figure S3 Heatmap of tumor and control cells in dataset GSE171957. In each cluster, N denotes the normal controls, and C denotes the tumor samples. **(A)** Comparison of BRCA1 wild-type ductal primary breast tumor and MCF10A; **(B)** comparison of TNBC and MCF10A tissues.

Supplementary Figure S4 Heatmap of 76NF2V and MCF10A cells in dataset GSE96860. MCF denotes MCF10A cells, and X76NF2V is 76NF2V cells.

Supplementary Figure S5 Heatmap of tumor tissue and normal control tissue from healthy donors. **(A)** ER+ BCa versus control tissue in dataset GSE45419; **(B)** HER2+ BCa versus control tissue in dataset GSE45419; **(C)** TNBC versus control tissue in dataset GSE45419; **(D)** HER2+ BCa versus control tissue in dataset GSE52194; E: TNBC versus control tissue in dataset GSE52194. In all legends in each cluster, N denotes normal tissue controls, and C denotes tumor tissues.

Supplementary Figure S6 Heatmap of tumor tissue (ER+) and adjacent normal tissue in dataset GSE58135. In all legends in each cluster, N denotes adjacent normal tissues, and C denotes tumor samples.

Supplementary Figure S7 Heatmap of tumor tissue (TNBC) and the adjacent normal tissue in dataset GSE58135. In all legends in each cluster, N denotes the adjacent normal tissues, and C denotes tumor samples.

Supplementary Figure S8 Heatmap of tumor tissue and adjacent normal tissue in dataset GSE103001. In the legends in each cluster, N denotes adjacent normal tissues, and C denotes tumor samples.

Supplementary Figure S9 Heatmap of tumor tissue and adjacent normal tissue in datasets GSE133998 and GSE183947. In all legends in each cluster, N denotes adjacent normal tissue, and C denotes tumor tissue.

Supplementary Figure S10 Heatmap of tumor tissue and adjacent normal tissue featuring metastatis in dataset GSE183947. TumorM indicates the tumor tissue with metastasis, TumorP indicates the tumor tissue without metastasis, NormalM indicates adjacent normal tissue with metastasis, and NormalP indicates adjacent normal tissue without metastasis.
