# Supplementary figures and images for: Screening and Identification of Human Endogenous Retrovirus-K mRNAs for Breast Cancer Through Integrative Analysis of Multiple Datasets
Source: Front Oncol. 2022 Feb 16;12:820883. doi: 10.3389/fonc.2022.820883 (PMC8900282; doi:10.3389/fonc.2022.820883)

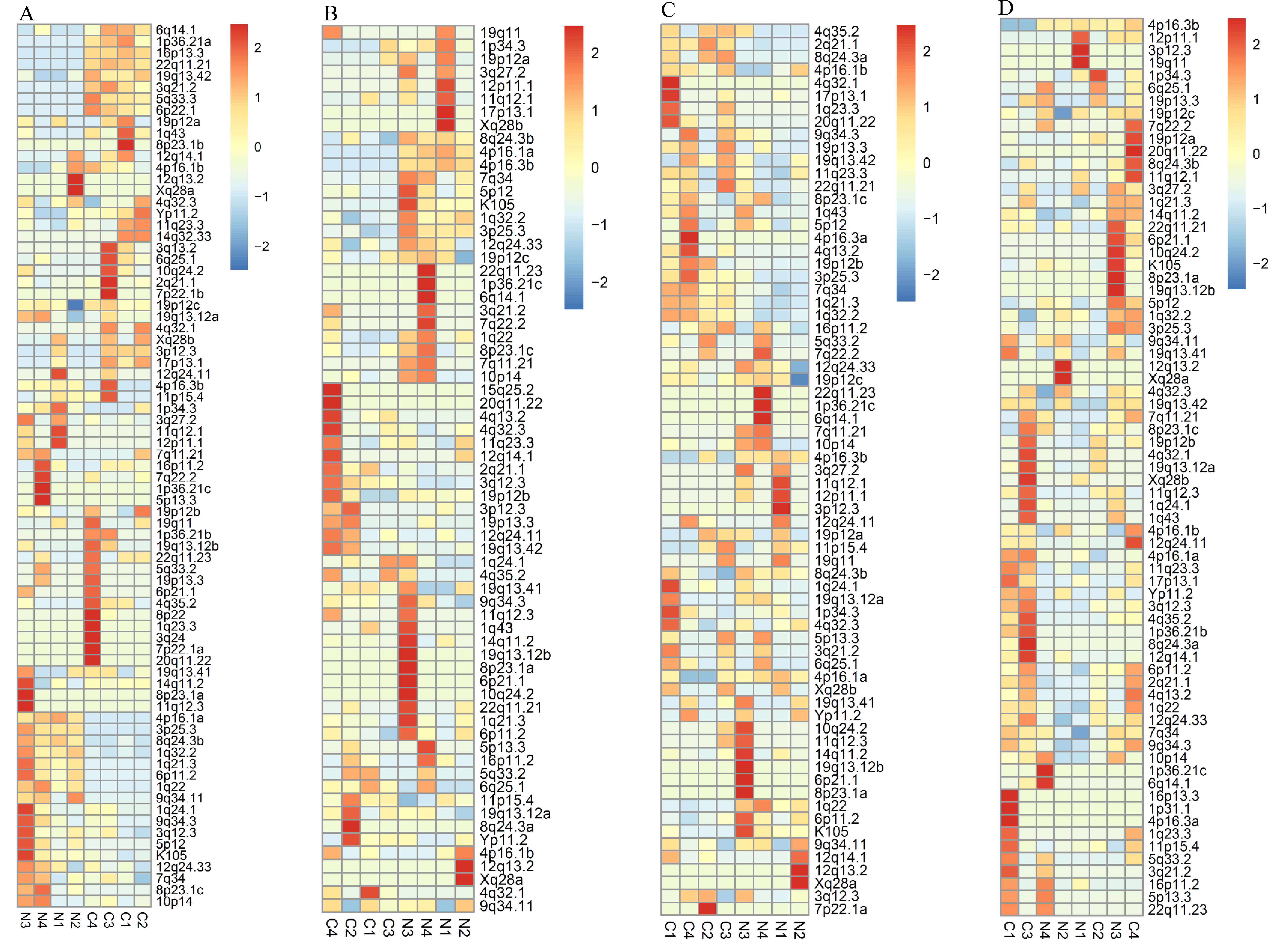

Supplement: Supplementary Figure S1 — Heatmap of tumor and control 76NF2V cells in dataset GSE96860. In all the legends in each cluster, N denotes normal controls, and C denotes tumor samples. (A) Comparison of AU565 and 76NF2V; (B) comparison of MB468 and 76NF2V; (C) comparison of MCF7 and 76NF2V; (D) comparison of MDAMB231 and 76NF2V. [file Image_1.tif]

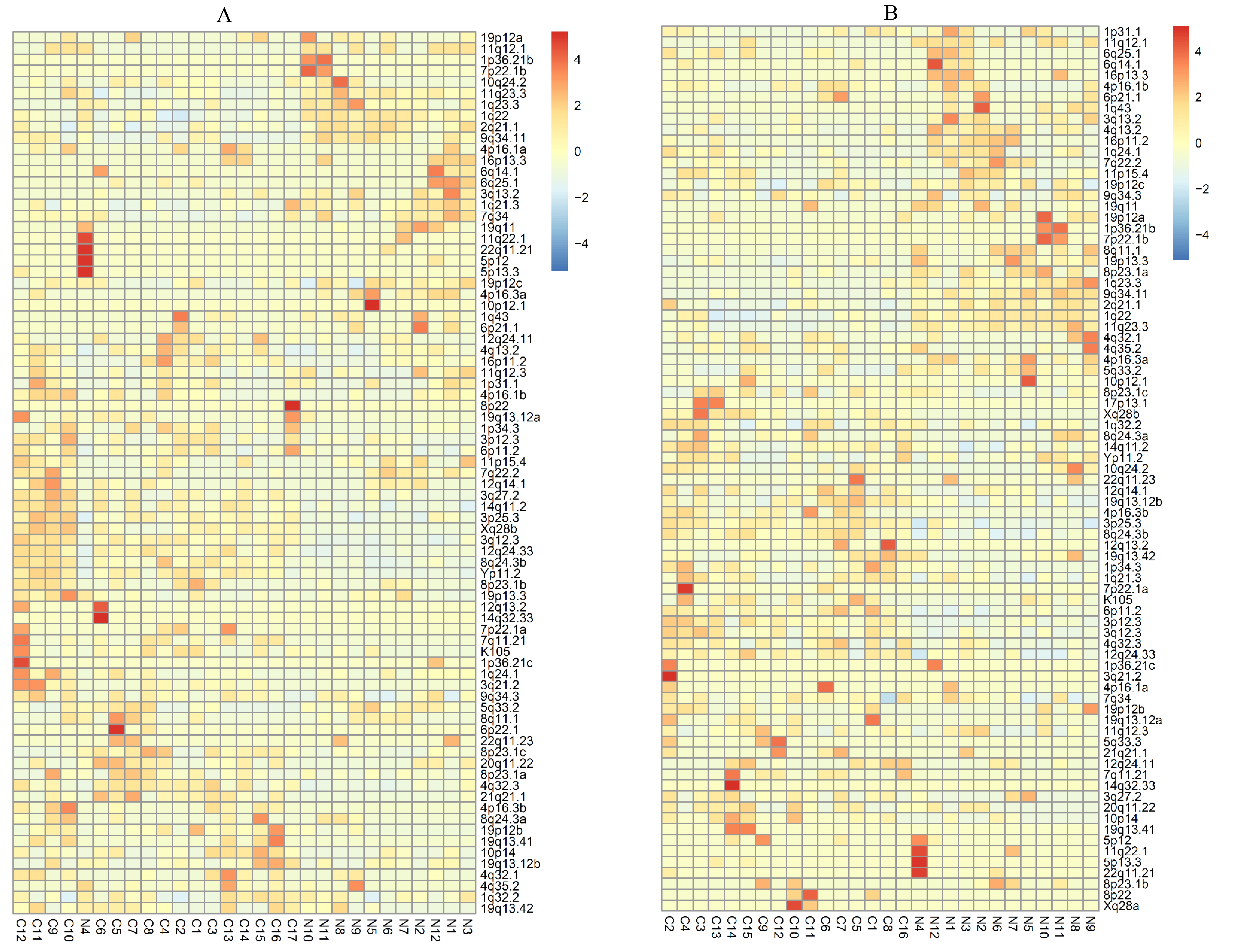

Supplement: Supplementary Figure S3 — Heatmap of tumor and control cells in dataset GSE171957. In each cluster, N denotes the normal controls, and C denotes the tumor samples. (A) Comparison of BRCA1 wild-type ductal primary breast tumor and MCF10A; (B) comparison of TNBC and MCF10A tissues. [file Image_3.tif]

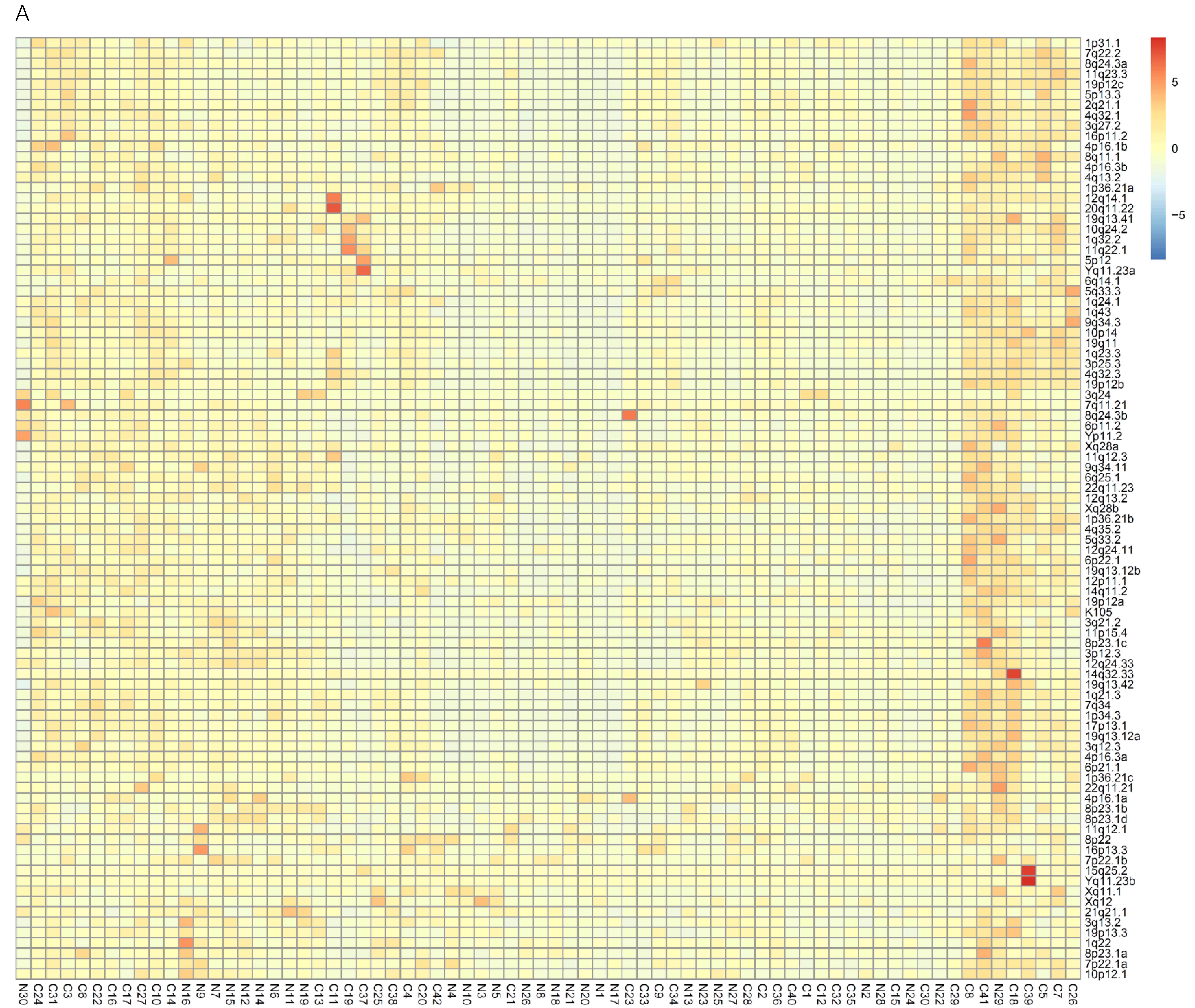

Supplement: Supplementary Figure S4 — Heatmap of 76NF2V and MCF10A cells in dataset GSE96860. MCF denotes MCF10A cells, and X76NF2V is 76NF2V cells. [file Image_4.jpeg]

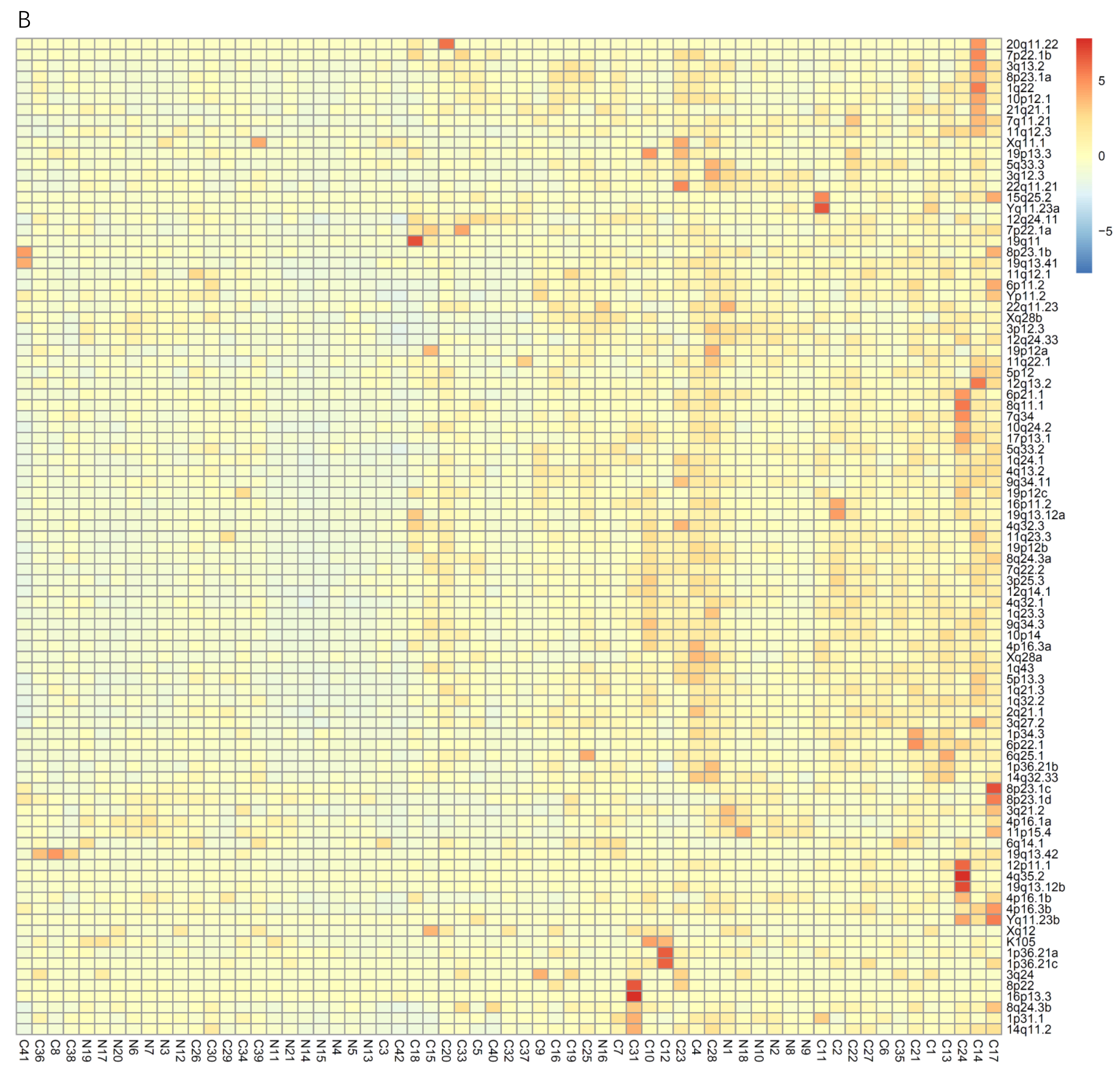

Supplement: Supplementary Figure S5 — Heatmap of tumor tissue and normal control tissue from healthy donors. (A) ER+ BCa versus control tissue in dataset GSE45419; (B) HER2+ BCa versus control tissue in dataset GSE45419; (C) TNBC versus control tissue in dataset GSE45419; (D) HER2+ BCa versus control tissue in dataset GSE52194; E: TNBC versus control tissue in dataset GSE52194. In all legends in each cluster, N denotes normal tissue controls, and C denotes tumor tissues. [file Image_5.jpeg]

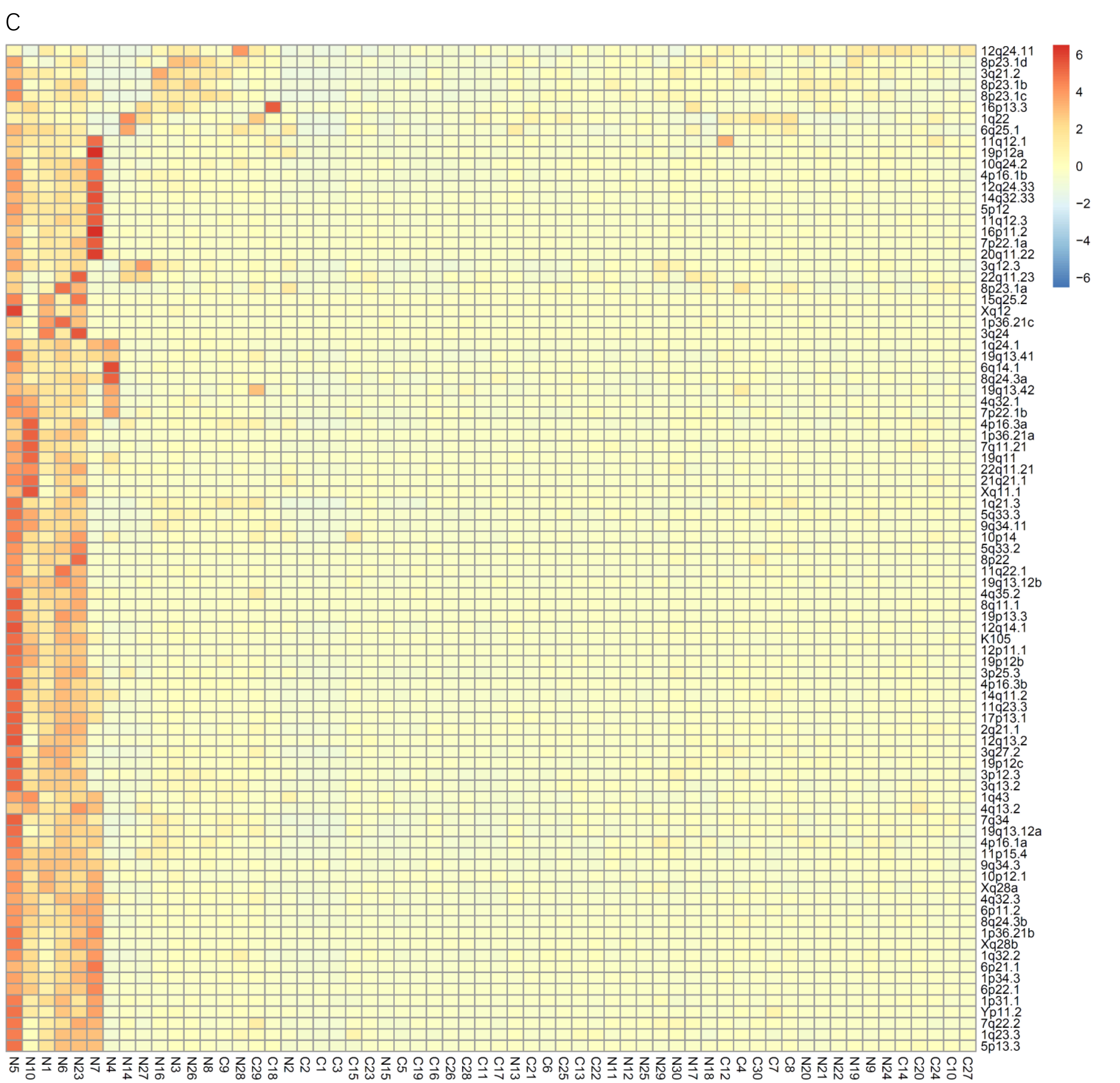

Supplement: Supplementary Figure S6 — Heatmap of tumor tissue (ER+) and adjacent normal tissue in dataset GSE58135. In all legends in each cluster, N denotes adjacent normal tissues, and C denotes tumor samples. [file Image_6.jpeg]

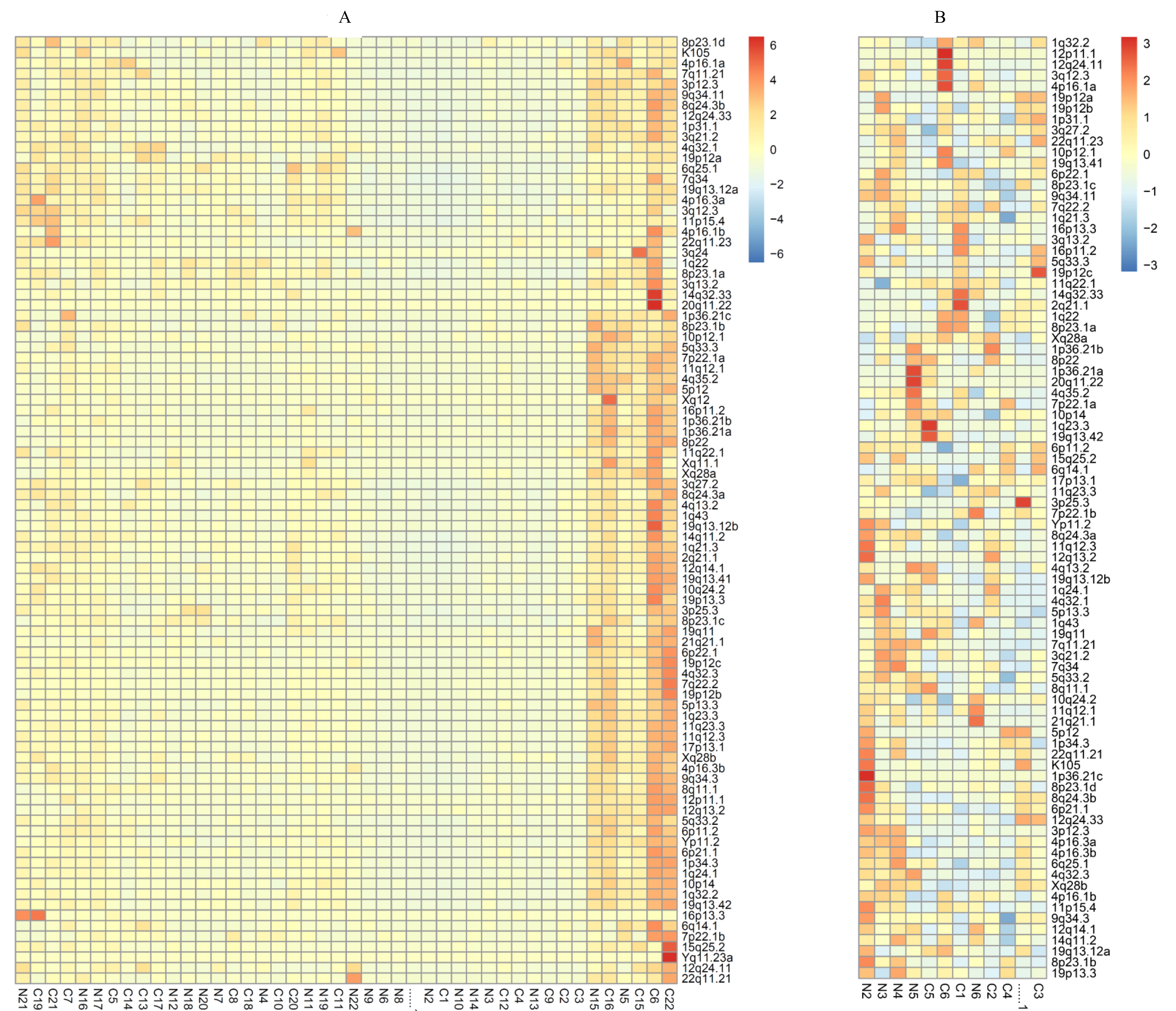

Supplement: Supplementary Figure S7 — Heatmap of tumor tissue (TNBC) and the adjacent normal tissue in dataset GSE58135. In all legends in each cluster, N denotes the adjacent normal tissues, and C denotes tumor samples. [file Image_7.jpeg]

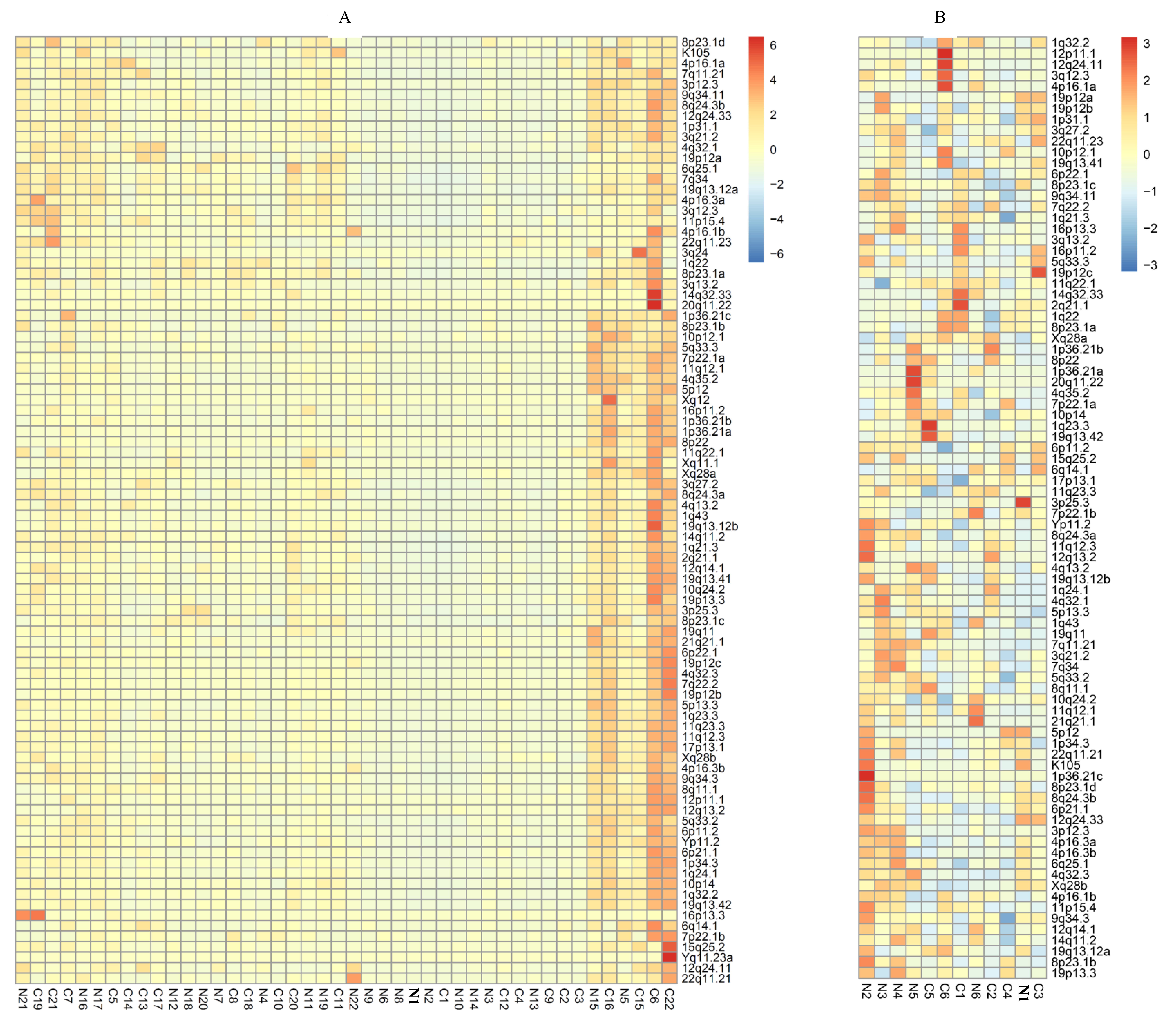

Supplement: Supplementary Figure S9 — Heatmap of tumor tissue and adjacent normal tissue in datasets GSE133998 and GSE183947. In all legends in each cluster, N denotes adjacent normal tissue, and C denotes tumor tissue. [file Image_9.jpeg]

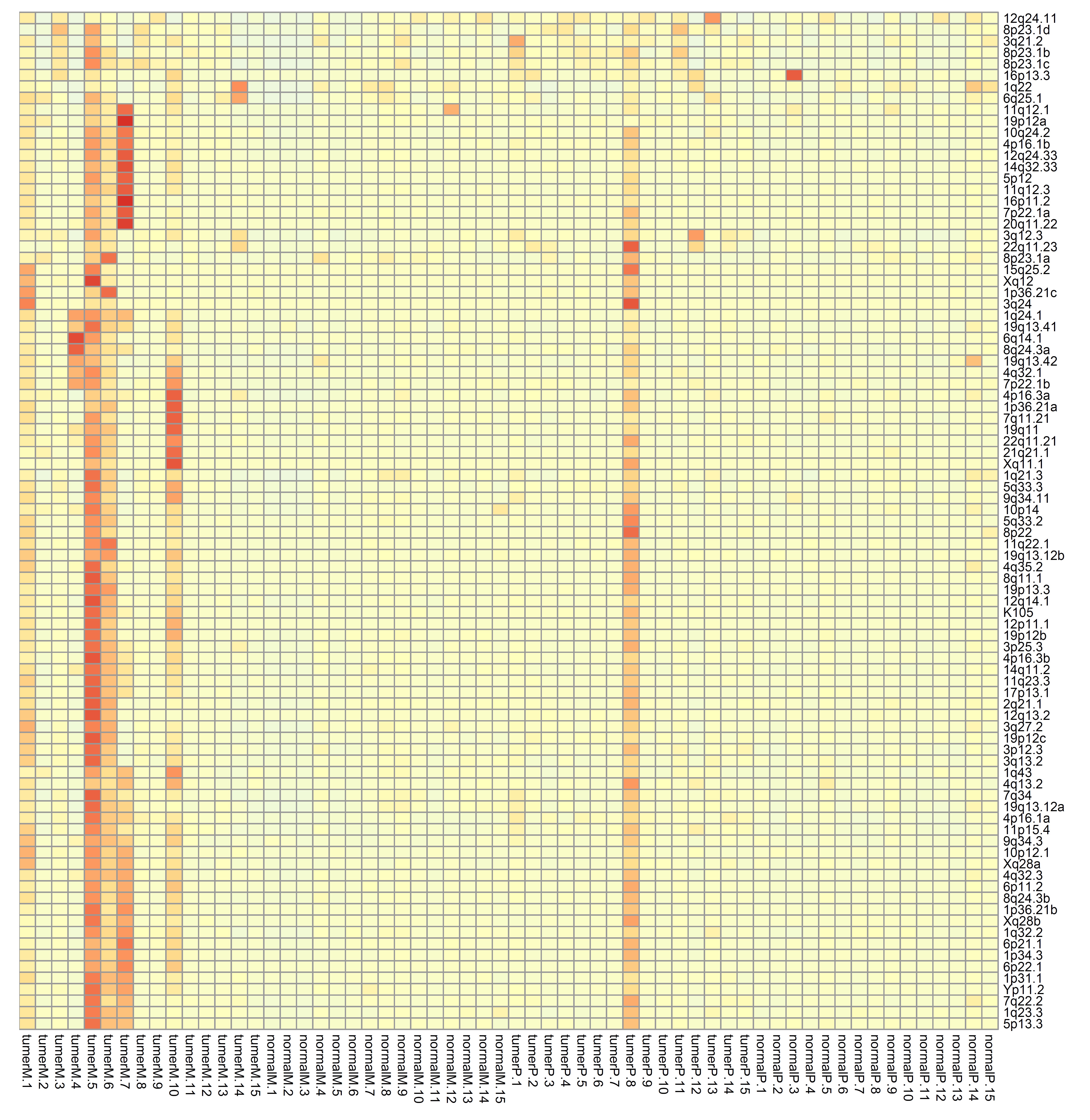

Supplement: Supplementary Figure S10 — Heatmap of tumor tissue and adjacent normal tissue featuring metastatis in dataset GSE183947. TumorM indicates the tumor tissue with metastasis, TumorP indicates the tumor tissue without metastasis, NormalM indicates adjacent normal tissue with metastasis, and NormalP indicates adjacent normal tissue without metastasis. [file Image_10.jpeg]
