# Supplementary material for: Screening and Identification of Human Endogenous Retrovirus-K mRNAs for Breast Cancer Through Integrative Analysis of Multiple Datasets
Source: Front Oncol. 2022 Feb 16;12:820883. doi: 10.3389/fonc.2022.820883 (PMC8900282; doi:10.3389/fonc.2022.820883)
Supplement: Supplementary file 11 [file Table_1.docx]

**Table S1 the character of nine expression profiling datasets downloaded from SRA/NCBI**

| Serious ID(Cancer tissue information) | Country | Platform | cDNA library | Controls samples | | Case sample | | filter condition | reference |
| --- | --- | --- | --- | --- | --- | --- | --- | --- | --- |
|  |  |  |  | number | Source | number | Source |  |  |
| GSE45419(primary breast tumors) | USA | Illumina Genome Analyzer IIx | paired-end | 8 | hyperplasia of breast | 8 ER+ BCa  8 HER2+ BCa  8 TNBC BCa | tissue | PE-Truseq2 | (1) |
| GSE111842  (primary tumors) | USA | Illumina HiSeq 2500 | paired-end | 6 | blood | 16 formalin-fixed, paraffin embedded primary BCa | blood | PE-Truseq3 | (2) |
| GSE96860 | USA | Illumina HiSeq 2000 | Singled-end | 4 76NF2V  4 MCF10A | cells | 4 MCF7  4 ZR751  4 MB361  4 UACC812  4 SKBR3  4 AU565  4 HCC1954  4 MB231  4 MB436  4 MB468  4 HCC1937 | cells | SE-Truseq3 | (3) |
| GSE171957 | USA | Illumina NextSeq 500 | Singled-end | 3 MCF10A | cells | 3 BRCA1-/- BCa | tissue | SE-Truseq3 | (4) |
|  |  |  |  |  |  | 3 TNBC BCa | tissue |  |  |
| GSE58135(primary tumors) | USA | Illumina HiSeq 2000 | paired-end | 51 | 21 adjacent to TNBC BCa  30 adjacent to ER+ BCa | 42 TNBC BCa  42 ER+ BCa | tissue | PE-Truseq3 | (5) |
| GSE52194(Primary Tumor) | USA | Illumina HiSeq 2000 | paired-end | 3 | normal human breast organoids | 6 TNBC BCa  5 HER2+ BCa | tissue | Truseq3 | (6) |
| GSE183947(including metastasis) | China | Illumina HiSeq 2000 | paired-end | 30 | Adjacent to BCa | 30 BCa | tissue | Truseq3 | missing |
| GSE133998(not mention) | China | HiSeq X Ten | paired-end | 6 | Adjacent to BCa | 6 BCa | tissue | Truseq3 | (7) |
| GSE103001(primary tumors) | Belgium | Illumina HiSeq 2000 | paired-end | 22 | Adjacent to BCa | 22 ER+ BCa | tissue | Truseq3 | (8) |

1. Kalari KR, Necela BM, Tang X, Thompson KJ et al. An integrated model of the transcriptome of HER2-positive breast cancer. PLoS One. 2013, 8(11):e79298.

2. Lang JE, Ring A, Porras T, Kaur P et al. RNA-Seq of Circulating Tumor Cells in Stage II-III Breast Cancer. Ann Surg Oncol 2018 Aug;25(8):2261-2270.

3. Franco HL, Nagari A, Malladi VS, Li W et al. Enhancer transcription reveals subtype-specific gene expression programs controlling breast cancer pathogenesis. Genome Res 2018 Feb;28(2):159-170.

4. Chappell K, Manna K, Washam CL, Graw S et al. Multi-omics data integration reveals correlated regulatory features of triple negative breast cancer. Mol Omics 2021 Oct 11;17(5):677-691.

5. Varley KE, Gertz J, Roberts BS, Davis NS et al. Recurrent read-through fusion transcripts in breast cancer. Breast Cancer Res Treat 2014 Jul;146(2):287-97.

6. Eswaran J, Cyanam D, Mudvari P, Reddy SD et al. Transcriptomic landscape of breast cancers through mRNA sequencing. Sci Rep 2012;2:264.

7. Xu X, Zhang J, Tian Y, Gao Y et al. CircRNA inhibits DNA damage repair by interacting with host gene. Mol Cancer 2020 Aug 24;19(1):128.

8. Wenric S, ElGuendi S, Caberg JH, Bezzaou W et al. Transcriptome-wide analysis of natural antisense transcripts shows their potential role in breast cancer. Sci Rep 2017 Dec 12;7(1):17452.

9. Wenric S, ElGuendi S, Caberg JH, Bezzaou W et al. Transcriptome-wide analysis of natural antisense transcripts shows their potential role in breast cancer. Sci Rep 2017 Dec 12;7(1):17452.
