# Supplementary material for: Screening and Identification of Human Endogenous Retrovirus-K mRNAs for Breast Cancer Through Integrative Analysis of Multiple Datasets
Source: Front Oncol. 2022 Feb 16;12:820883. doi: 10.3389/fonc.2022.820883 (PMC8900282; doi:10.3389/fonc.2022.820883)
Supplement: Supplementary file 12 [file Table_2.docx]

Table S2 the number of differentially expressed HERVKs identified from each dataset

| series accession | platform | sample size | total differentially expressed genes | Upregulated genes | Downregulated genes |
| --- | --- | --- | --- | --- | --- |
| GSE96860 | GPL11154 | total: 8 | 2 | 1 | 1 |
|  |  | cell(76NF2V): 4 |  |  |  |
|  |  | cell(MCF7): 4 |  |  |  |
| GSE96860 | GPL11154 | total: 8 | 6 | 4 | 2 |
|  |  | cell(76NF2V): 4 |  |  |  |
|  |  | cell(ZR751): 4 |  |  |  |
| GSE96860 | GPL11154 | total: 8 | 6 | 5 | 1 |
|  |  | cell(76NF2V): 4 |  |  |  |
|  |  | cell(UACC812): 4 |  |  |  |
| GSE96860 | GPL11154 | total: 8 | 8 | 6 | 2 |
|  |  | cell(76NF2V): 4 |  |  |  |
|  |  | cell(MDA-MB361): 4 |  |  |  |
| GSE96860 | GPL11154 | total: 8 | 8 | 6 | 2 |
|  |  | cell(76NF2V): 4 |  |  |  |
|  |  | cell(AU565): 4 |  |  |  |
| GSE96860 | GPL11154 | total: 8 | 2 | 0 | 2 |
|  |  | cell(76NF2V): 4 |  |  |  |
|  |  | cell(HCC1954): 4 |  |  |  |
| GSE96860 | GPL11154 | total: 8 | 7 | 5 | 2 |
|  |  | cell(76NF2V): 4 |  |  |  |
|  |  | cell(SKBR3): 4 |  |  |  |
| GSE96860 | GPL11154 | total: 8 | 4 | 3 | 1 |
|  |  | cell(76NF2V): 4 |  |  |  |
|  |  | cell(HCC1037): 4 |  |  |  |
| GSE96860 | GPL11154 | total: 8 | 4 | 3 | 1 |
|  |  | cell(76NF2V): 4 |  |  |  |
|  |  | cell(MB468): 4 |  |  |  |
| GSE96860 | GPL11154 | total: 8 | 3 | 3 | 0 |
|  |  | cell(76NF2V): 4 |  |  |  |
|  |  | cell(MDA-MB231): 4 |  |  |  |
| GSE96860 | GPL11154 | total: 8 | 5 | 4 | 1 |
|  |  | cell(76NF2V): 4 |  |  |  |
|  |  | cell(MDA-MB436): 4 |  |  |  |
| GSE96860 | GPL11154 | total: 8 | 9 | 0 | 9 |
|  |  | cell(MCF10A): 4 |  |  |  |
|  |  | cell(MCF7): 4 |  |  |  |
| GSE96860 | GPL11154 | total: 8 | 7 | 1 | 6 |
|  |  | cell(MCF10A): 4 |  |  |  |
|  |  | cell(ZR751): 4 |  |  |  |
| GSE96860 | GPL11154 | total: 8 | 7 | 2 | 5 |
|  |  | cell(MCF10A): 4 |  |  |  |
|  |  | cell(UACC812): 4 |  |  |  |
| GSE96860 | GPL11154 | total: 8 | 14 | 2 | 12 |
|  |  | cell(MCF10A): 4 |  |  |  |
|  |  | cell(MDA-MB361): 4 |  |  |  |
| GSE96860 | GPL11154 | total: 8 | 9 | 2 | 7 |
|  |  | cell(MCF10A): 4 |  |  |  |
|  |  | cell(AU565): 4 |  |  |  |
| GSE96860 | GPL11154 | total: 8 | 11 | 0 | 11 |
|  |  | cell(MCF10A): 4 |  |  |  |
|  |  | cell(HCC1954): 4 |  |  |  |
| GSE96860 | GPL11154 | total: 8 | 8 | 0 | 8 |
|  |  | cell(MCF10A): 4 |  |  |  |
|  |  | cell(SKBR3): 4 |  |  |  |
| GSE96860 | GPL11154 | total: 8 | 6 | 0 | 6 |
|  |  | cell(MCF10A): 4 |  |  |  |
|  |  | cell(HCC1037): 4 |  |  |  |
| GSE96860 | GPL11154 | total: 8 | 10 | 2 | 8 |
|  |  | cell(MCF10A): 4 |  |  |  |
|  |  | cell(MB468): 4 |  |  |  |
| GSE96860 | GPL11154 | total: 8 | 4 | 2 | 2 |
|  |  | cell(MCF10A): 4 |  |  |  |
|  |  | cell(MDA-MB231): 4 |  |  |  |
| GSE96860 | GPL11154 | total: 8 | 3 | 0 | 3 |
|  |  | cell(MCF10A): 4 |  |  |  |
|  |  | cell(MDA-MB436): 4 |  |  |  |
| GSE171957 | GPL18573 | total: 8 | 15 | 1 | 14 |
|  |  | cell(MCF10A): 4 |  |  |  |
|  |  | cell(HCC1937): 4 |  |  |  |
| GSE171957 | GPL18573 | total: 8 | 11 | 6 | 5 |
|  |  | cell(MCF10A): 4 |  |  |  |
|  |  | cell(MDA-231): 4 |  |  |  |
| GSE45419 | GPL10999 | total: 16 | 15 | 15 | 0 |
|  |  | Benign cell lines: 8 |  |  |  |
|  |  | tumor(ER+): 8 |  |  |  |
| GSE45419 | GPL10999 | total: 16 | 11 | 11 | 0 |
|  |  | Benign cell lines: 8 |  |  |  |
|  |  | tumor(HER2+): 8 |  |  |  |
| GSE45419 | GPL10999 | total: 16 | 8 | 7 | 1 |
|  |  | Benign cell lines: 8 |  |  |  |
|  |  | tumor(TNBC): 8 |  |  |  |
| GSE58135 | GPL11154 | total: 72 | 0 | 0 | 0 |
|  |  | adjacent: 30 |  |  |  |
|  |  | tumor(ER+): 42 |  |  |  |
| GSE58135 | GPL11154 | total: 63 | 3 | 3 | 0 |
|  |  | adjacent: 21 |  |  |  |
|  |  | tumor(TNBC): 42 |  |  |  |
| GSE103001 | GPL11154 | total: 44 | 2 | 2 | 0 |
|  |  | adjacent: 22 |  |  |  |
|  |  | tumor: 22 |  |  |  |
| GSE133998 | GPL20795 | total: 12 | 1 | 0 | 1 |
|  |  | adjacent: 6 |  |  |  |
|  |  | tumor: 6 |  |  |  |
| GSE183947 | GPL11154 | total: 60 | 0 | 0 | 0 |
|  |  | adjacent normal: 30 |  |  |  |
|  |  | tumor: 30 |  |  |  |
