# Supplementary material for: Screening and Identification of Human Endogenous Retrovirus-K mRNAs for Breast Cancer Through Integrative Analysis of Multiple Datasets
Source: Front Oncol. 2022 Feb 16;12:820883. doi: 10.3389/fonc.2022.820883 (PMC8900282; doi:10.3389/fonc.2022.820883)
Supplement: Supplementary file 14 [file Table_4.docx]

Table S4 The expression of 17p13.1 and TP53 in dataset GSE45419

| Name | Normal | Normal | Normal | Normal | Normal | Normal | Normal | Normal | BCa | BCa | BCa | BCa | BCa | BCa | BCa | BCa |
| --- | --- | --- | --- | --- | --- | --- | --- | --- | --- | --- | --- | --- | --- | --- | --- | --- |
| 17P13.1 | 0 | 0 | 0 | 0 | 0 | 0 | 0 | 0 | 0.39 | 0.01 | 0 | 0.029 | 0.01 | 0.01 | 0 | 0 |
| TP53 | 41.66 | 41.66 | 42.83 | 38.16 | 44.01 | 41.02 | 33.45 | 39.70 | 17.308 | 7.45 | 32.27 | 36.95 | 28.16 | 26.77 | 135.39 | 46.10 |
